# Establishing clinically meaningful within-individual improvement thresholds for eight patient-reported outcome measures in people with relapsing-remitting multiple sclerosis

**DOI:** 10.1186/s41687-023-00594-8

**Published:** 2023-07-04

**Authors:** Nupur Greene, Stéphane Quéré, Denise P. Bury, Flora Mazerolle, Manal M’Hari, Angély Loubert, Antoine Regnault, Keiko Higuchi

**Affiliations:** 1 Sanofi, 450 Water Street, Cambridge, MA USA; 2 Modus Outcomes, A Division of THREAD, Lyon, France

**Keywords:** Teriflunomide, Relapsing-remitting multiple sclerosis (RRMS), Patient-reported outcome (PRO), Validation, Within-patient change threshold

## Abstract

**Background:**

As disease-modifying therapies do not reverse the course of multiple sclerosis (MS), assessment of therapeutic success involves documenting patient-reported outcomes (PROs) concerning health-related quality of life, disease and treatment-related symptoms, and the impact of symptoms on function. Interpreting PRO data involves going beyond statistical significance to calculate within-patient meaningful change scores. These thresholds are needed for each PRO in order to fully interpret the PRO data. This analysis of PRO data from the PROMiS AUBAGIO study, which utilized 8 PRO instruments in teriflunomide-treated relapsing-remitting MS (RRMS) patients, was designed to estimate clinically meaningful within-individual improvement thresholds in the same manner, for 8 PRO instruments.

**Results:**

The analytical approach followed a triangulation exercise that considered results from anchor- and distribution-based methods and graphical representations of empirical cumulative distribution functions in PRO scores in groups defined by anchor variables. Data from 8 PRO instruments (MSIS-29 v2, FSMC, MSPS, MSNQ, TSQM v1.4, PDDS, HRPQ-MS v2, and HADS) were assessed from 434 RRMS patients. For MSIS-29 v2, FSMC, MSPS, and MSNQ total scores, available anchor variables enabled both anchor- and distribution-based methods to be applied. For instruments with no appropriate anchor available, distribution-based methods were applied. A recommended value for meaningful within-individual improvement was defined by comparing mean change in PRO scores between participants showing improvement of one or two categories in the anchor variable or those showing no change. A “lower bound” estimate was calculated using distribution-based methods. An improvement greater than the lower-bound estimate was considered “clinically meaningful”.

**Conclusion:**

This analysis produced estimates for assessing meaningful within-individual improvements for 8 PRO instruments used in MS studies. These estimates should be useful for interpreting scores and communicating study results and should facilitate decision-making by regulatory and healthcare authorities where these 8 PROs are commonly employed.

**Supplementary Information:**

The online version contains supplementary material available at 10.1186/s41687-023-00594-8.

## Background

Multiple sclerosis (MS) is a chronic immune-mediated disease affecting the central nervous system [1, 2]. It has been estimated that 2.8 million people had MS in 2020, equivalent to ~ 35.9 per 100,000 population, which has risen by 30%, globally, since 2013 [3], with females and young adults disproportionately affected [3, 4]. As such, MS is the leading cause of nontraumatic neurological disability in young adults, with a mean age at diagnosis (globally) of 32 years [3, 5]. Being a life-long disease, effective disease monitoring and management are critical ideally from symptom onset. The cornerstone of MS management is with disease-modifying therapies (DMTs) [6], which decrease frequency, duration, and severity of relapses in people with relapsing forms of MS (relapsing-remitting MS [RRMS]). Certain DMTs can also reduce disease progression [7, 8]; however, there is no treatment that can reverse the disease course of MS [5, 9]. Therefore, treatment also focuses on improving or maintaining health-related quality of life (HRQOL), minimizing the impact of disability and side-effects of treatment, and maximizing wellness [10]. Consequently, patient-reported outcomes (PROs), where patients self-report and describe their perceived health status, function, and/or experiences, are especially relevant in MS, as they provide individualized perspectives about health experience and treatment outcomes [10] during periods of worsening or stable disease [11]. Indeed, PROs are recognized as having an increasingly important role in MS clinical research and within real-world clinical practice [12, 13].

Both MS-specific and generic PRO instruments are beneficial in evaluating HRQOL in people with MS and in assessing the impact of MS on function; hence, a combined approach using both generic and MS-specific PROs is often recommended [10]. Given their importance, interpretation of PRO scores is critical to enable communication about PRO results to regulators, patients, physicians, and healthcare providers alike and to inform decision-making by regulatory boards and healthcare authorities [14]. However, there is currently little standardization in how PRO measures are scored or presented, and there is some confusion reported regarding the thresholds used to assess clinical significance [10]. The number of PRO measures used in MS trials makes comparison difficult [12]. Interpretation and communication of PRO results is challenging given that many MS PROs are routinely used in clinical trials but they lack established meaningful within-patient change thresholds, which are needed to interpret responder and time-to-event analyses [11]. Without commonly derived thresholds in place across a range of frequently used instruments, application of PROs for the decision-making process for regulatory or reimbursement agency reviews for MS medications could limit their utility, just when their importance is increasingly recognized [15, 16]. Consequently, further information is needed to help validate the use of multiple PROs within the MS healthcare arena and to summarize clinical meaningfulness from these different assessments.

The Prospective, Non-Interventional Trial to Analyze Patient-Reported Outcomes in MS Patients Treated with Teriflunomide (AUBAGIO^®^) in Routine Clinical Practice (PROMiS AUBAGIO study [TERIFL07766]) was a prospective, noninterventional study in US participants with RRMS treated with oral teriflunomide in routine clinical practice. Eight PROs were utilized in the PROMiS AUBAGIO study to evaluate the impact of RRMS on HRQOL, fatigue, functional performance, work capacity, daily activities, cognitive impairment, anxiety and depression, and satisfaction with treatment. This analysis of PROMiS AUBAGIO data was designed to estimate meaningful within-individual improvement thresholds for eight PRO instruments used in this study. It was hoped that this would enable better interpretation of improvement scores for these eight PRO instruments in past, current, and future MS studies.

## Methods

### Participants

The PROMiS AUBAGIO study included participants aged ≥ 18 years with RRMS treated with oral teriflunomide in routine clinical practice in the US. The primary objective was to describe changes in HRQOL in RRMS participants who initiated treatment with teriflunomide, using scores from eight MS-specific and generic PRO instruments. All participants provided written, informed consent prior to entry into the study. It was planned that 740 patients would be recruited from the MS ONE-TO-ONE program such that, accounting for attrition, ~ 500 participants would complete PRO assessments at Month 12. This analysis was based on data from 434 RRMS participants who signed the electronic informed consent, had ≥ 1-day study medication exposure, and baseline and ≥ 1 post-baseline PRO assessment. This cohort is defined as the full-analysis set (FAS).

### PRO assessments

A total of eight unique PRO instruments were administered to participants during the PROMiS AUBAGIO study; these instruments cover a variety of MS-specific symptoms, including HRQOL, fatigue, functional performance, work capacity, daily activities, cognitive impairment, anxiety and depression, and satisfaction with treatment. A summary of PRO scores and schedule of assessments is given in Table 1. All data were collected electronically through the online portal at baseline and subsequent months depending on the instrument (Table 1). All data were directly entered by study participants. All participant health information was encrypted. All PROs were selected based on their context of use and ability to capture the concepts most relevant to patients/caregivers in the RRMS population. Each PRO had evidence in the literature for its reliability, as outlined below.

**Table 1:** Patient-reported outcome instruments administered during the PROMiS AUBAGIO study

Instrument	PRO scores			Assessment schedule (# of assessments)
	Score	Number of items	Score range	
*Instruments assessed by anchor-based and distribution-based analysis:*				
MSIS-29 v2	Physical impact	20	0–100	BL, M4, M8, M12 (n = 4)
	Psychological impact	9		
FSMC	Physical fatigue	10	10–50	BL, M2, M5, M8, M11 (n = 5)
	Cognitive fatigue	10	10–50	
	Total	20	20–100	
MSPS	Total	8	0–40	BL, M4, M8, M12 (n = 4)
MSNQ	Total	15	0–60	BL, M3, M6, M9, M12 (n = 5)
*Instruments assessed by distribution-based analysis only:*				
TSQM v1.4	Side effects	4	0–100	BL, M1, M4, M7, M10 (n = 5)
	Effectiveness	3	0–100	
	Convenience	3	0–100	
	Global satisfaction	3	0–100	
PDDS		1	0–8	BL, M6, M12 (n = 3)
HRPQ-MS v2		9	Open ended/multiple choice	BL, M1, M5, M8, M11 (n = 5)
HADS	Anxiety	7	0–21	BL, M3, M6, M9, M12 (n = 5)
	Depression	7	0–21	

*BL* Baseline; *FSMC* Fatigue Scale for Motor and Cognitive Functions; *HADS* Hospital Anxiety and Depression Scale; *HRPQ-MS v2* Health-Related Productivity Questionnaire in Multiple Sclerosis version 2; *M* Month x; *MSIS-29 v2* Multiple Sclerosis Impact Scale 29 items version 2; *MSNQ* Multiple Sclerosis Neuropsychological Screening Questionnaire; *MSPS* Multiple Sclerosis Performance Scale; *PDDS* Patient-Determined Disease Steps; *PRO* Patient-reported outcome; *TSQM v1.4* Treatment Satisfaction Questionnaire with Medication version 1.4

#### Multiple Sclerosis Impact Scale 29 items version 2 (MSIS-29 ﻿v2﻿)﻿

The MSIS-29 v2 is a PRO instrument developed to evaluate specific physical and psychological impact of MS from the patient’s perspective; the instrument comprises 29 items grouped into physical impact (20 items) and psychological impact (9 items) scores [17, 18]. Participants respond to each item regarding the condition’s impact on their daily life during the last two weeks and select an answer on a five-point Likert scale that strongly represented their status. The patient’s scores on the two subscales can be summed and converted to a measure between 0 and 100, where higher scores indicate a greater physical and psychological disease impact (worse health status [17]). The current study used each scale separately. Test–retest reliability has been shown to be high (r = 0.65–0.90) [17]. The administration schedule for the scale is given in Table 1.

#### Fatigue Scale for Motor and Cognitive Functions (FSMC)

The FSMC was developed to evaluate MS-related motor and cognitive fatigue, comprising 20 items that are grouped into a physical fatigue score (10 items), cognitive fatigue score (10 items), and combined total score (20 items) [19]. Each dimension score ranges from 10 to 50, and the total score from 20 to 100, with a higher total score indicating more severe fatigue. Internal consistency (Cronbach’s alpha > 0.91) as well as test–retest reliability (r > 0.80) has been shown to be high [19]. The administration schedule for the scale is given in Table 1.

#### Multiple Sclerosis Performance Scale (MSPS)

The MSPS was developed to evaluate MS-associated disability overall and in different dimensions and comprises 8 performance scales (mobility, hand function, vision, fatigue, cognition, bowel/bladder, sensory, spasticity), and a total score [20]. Each individual scale ranges from 0 (normal) to 5 (total disability), and the total score ranges from 0 (normal) to 40 (total disability), with higher individual and total scores indicating more disability. Spearman’s rank correlations assessing convergent construct validity range between 0.55 and 0.78 [20]. The administration schedule for the scale is given in Table 1.

#### Multiple Sclerosis Neuropsychological Screening Questionnaire (MSNQ﻿)

The MSNQ was developed to identify neuropsychological impairment in MS and includes a patient and an informant/caregiver version [21, 22]. The patient version of the MSNQ, which comprises 15 items, was used in this study. Patients rate themselves from 0 (never; does not occur) to 4 (very often; very disrupted) on specific cognitive and behavioral problems that may arise in daily life. These 15 items are grouped into a total score ranging from 0 to 60, where higher scores indicate increased neuropsychological impairment or depressive disorder. Cronbach's alpha coefficient for the patient MSNQ is 0.93, and the PRO has been strongly correlated with a more general cognitive complaints questionnaire [22]. The administration schedule for the scale is given in Table 1.

#### Treatment Satisfaction Questionnaire with Medication version 1.4 (TSQM v1.4﻿)

The TSQM (version-1.4) was developed as a generic instrument to evaluate patients’ satisfaction with medication and comprises 14 items grouped into side-effects score (4 items), effectiveness score (3 items), convenience score (3 items), and global satisfaction score (3 items) [23, 24]. The remaining question is a filter item [24]. Each score ranges from 0 to 100, where higher scores indicate greater satisfaction with the different aspects of medication. Internal consistency reliability in MS patients is reportedly high (Cronbach’s α > 0.90) [25]. The administration schedule for the scale is given in Table 1.

#### Patient-Determined Disease Steps (PDDS﻿)

The PDDS scale was developed to evaluate MS-associated disability overall and in different dimensions, and is a single ordinal item ranging from 0 (normal) to 8 (bedridden), with a higher score indicating more disability [26, 27]. PDDS had a strong [27], albeit not perfect [26], correlation with the Expanded Disability Status Scale (EDSS). In translation, PDDS has shown excellent test–retest reliability [28–30]. The administration schedule for the scale is given in Table 1.

#### Health-Related Productivity Questionnaire in Multiple Sclerosis version 2 (HRPQ-MS v2﻿)

The HRPQ-MS v2 was developed from the original HRPQ instrument [31] to evaluate health-related productivity in people being treated for MS [32, 33]. This tool comprises 9 open and multiple-choice questions that quantify hours of employment-related lost productivity and household-related lost productivity. HRPQ has good construct and criterion validity with the Work Productivity and Activity Impairment Questionnaire (WPAI) (Pearson’s r ≥ 0.6, *P* < 0.05) [32]. The administration schedule for the scale is given in Table 1.

#### Hospital Anxiety and Depression Scale (HADS﻿)

The HADS is a generic self-administered questionnaire to evaluate states of anxiety and depression and comprises 14 items that are grouped into an anxiety (7 items) and depression score (7 items) [34–36]. Each score ranges from 0 (normal) to 21 (abnormal), with higher scores indicating increased anxiety or depression. HADS Anxiety and Depression scales have acceptable internal consistency reliability as measured by Cronbach's alpha (0.86, 0.82, respectively) in people with MS [37]. In addition, test–retest reliability was 0.83 for both HADS Anxiety and HADS Depression scales [37]. The administration schedule for the scale is given in Table 1.

### Statistical analysis

#### Data analysis

Analysis was performed using SAS software version 9.4 (SAS Institute Inc, Cary, NC, USA). Continuous variables are described by frequency, mean, standard deviation (SD), median, [minimum, maximum], and number of missing values. Categorical variables are described by frequency and percentages, with missing data included in calculation of percentages. Missing item responses within a PRO questionnaire were managed in the creation of PRO scores as specified by the developers of the instruments. Analyses were conducted on the FAS.

#### Anchor-based analyses

Anchor-based methods are the primary approach for determining a meaningful within-individual change threshold, as recommended by the US Food and Drug Administration (FDA) [15], and were used for the MSIS-29, FSMC, MSPS, and MSNQ scales. Available variables used as potential anchors were PDDS for MSIS-29 (physical and psychological impact scores); MSPS (total score); MSPS fatigue item for FSMC (physical fatigue, mental fatigue, total scores); and MSPS cognition item for MSNQ (total score). Time period assessed was change from baseline to Month 12, with the exception of FSMC, for which baseline to Month 11 was used.

Correlation between each anchor variable and the corresponding PRO score and between change in each anchor variable and change in the corresponding PRO score was estimated using Spearman’s rank-order correlation coefficients. Change in PRO scores was described in the groups defined according to the change in anchor variables over the same time period.

Receiver-operating characteristic (ROC) curves were plotted to characterize the separation of groups defined according to various dichotomous categorizations of the anchor variables by the change in corresponding PRO score; from these ROC curves, change in PRO score that maximized the separation between dichotomous categorizations of the anchor variable was estimated by the smallest sum of squares of 1-sensitivity and 1-specificity [38].

#### Empirical cumulative distribution function (eCDF) and probability density function (PDF)

eCDF and PDF of change in MSIS-29, FSMC, MSPS, and MSNQ scores were plotted over the specified time periods according to groups defined by the potential anchors. Graphical representations of eCDFs of change in PRO scores in groups were defined by the anchor variables (Additional file 1: Fig. S1). For the MSIS-29 v2 and FSMC scores, as well as MSPS and MSNQ total scores, anchor variables (ie, variables assessing similar concepts) were available for both anchor-based and distribution-based methods and, therefore, could be applied.

#### Distribution-based analyses

Distribution-based methods were used to generate additional supportive data to define meaningful within-individual change in all PRO scores, including PROs covered by anchor-based methods. For each score, values corresponding to previously defined effect size (ES) thresholds [39] were calculated using the score SD at baseline: 0.2SD, 0.5SD, and 0.8SD. The standard error of measurement (SEM) of each score was then calculated; SEM of a score was defined from the score SD and its reliability coefficient (r) $\left( {SD \times \sqrt {\left( {1 - r} \right)} } \right)$, which was estimated using Cronbach’s coefficient alpha calculated at baseline.

#### Triangulation and PRO responder analysis

The results of the analyses were triangulated to define a reference value and a conservative value for the clinically meaningful within-individual change in MSIS-29, FSMC, MSPS, MSNQ, and a reference value for the clinically meaningful within-individual change for TSQM, PDDS, HRPQ-MS, and HADS scores. Results from the anchor-based methods represent meaningful within-individual *improvement* only, while results from the distribution-based methods are based on the score distribution hence have no direction and represent meaningful *change*. The reference value was established as change in PRO score of participants who showed an improvement of one or two categories in the corresponding anchor variable for PRO scores for which anchor-based methods were applied. For PRO scores in which only distribution-based methods were applied, the reference value was defined by 0.5SD at baseline.

For establishing a range of meaningful within-patient improvement, a “conservative” value was defined based on anchor-based and distribution-based methods. This value was defined to acknowledge the uncertainty of the estimate and provide a value that can be considered a “worst-case scenario”. The values obtained by the analyses were compared with the minimum detectable change $\left( {1.96 \times \sqrt 2 \times SEM} \right)$﻿, which is the smallest change that is above measurement error [40]. The estimated meaningful within-individual improvement value was then evaluated to determine whether it can be observed in practice; if not, the closest greater observable value was used as the meaningful within-individual improvement value. We considered any improvement greater than the lower-bound estimate using the “worst case scenario” approach to be “clinically meaningful.”

A responder was defined as a participant with a change in score from baseline greater than the clinically meaningful within-individual improvement value. The percentage responder analysis was calculated at Month 10 for TSQM v1.4 scores, Month 11 for FSMC and HRPQ-MS v2 scores, and Month 12 for all other scores.

## Results

### Patient characteristics

Baseline characteristics and PRO scores in the FAS (n = 434) are summarized in Table 2. Participants were heterogeneous in terms of both demographic and clinical characteristics. The majority of participants were female (77%) and white (86%), and the mean ± SD age was 50 ± 11 years. Time since first diagnosis of MS was 11 ± 10 years, with 25% of participants diagnosed for less than 3 years, and 25% for more than 17 years. The majority of patients (62%) had experienced ≥ 1 relapse in the past year.

**Table 2:** Baseline characteristics and PRO scores

Variable	Total (N = 434)
Age, years, mean (SD)	50.2 (10.5)
Female gender, n (%)	336 (77.4)
Race, n (%)	
White	375 (86.4)
Black	36 (8.3)
Hispanic	20 (4.6)
Other^a^	3 (0.7)
Prior MS therapy, n (%)	353 (81.3)
Time since first diagnosis of MS^b^, years, mean (SD)	11.2 (9.9)
Time since last MS relapse^c^, months, mean (SD)	31.1 (50.5)
Number of MS relapses in the last year, mean (SD)	1.5 (2.1)
0, n (%)	165 (38.0)
1, n (%)	122 (28.1)
2, n (%)	64 (14.7)
≥ 3, n (%)	83 (19.1)

		Mean (SD)	Median (min, max)
*Multi-item PRO scores*			
MSIS-29 v2	Physical impact (n = 429)	36.6 (25.4)	33.3 (0, 100)
	Psychological impact (n = 429)	41.0 (24.8)	40.7 (0, 100)
FSMC	Physical fatigue (n = 429)	31.8 (11.1)	34.0 (10, 50)
	Cognitive fatigue (n = 429)	29.3 (11.2)	30.0 (10, 50)
	Total score (n = 429)	61.1 (21.6)	64.0 (20, 100)
MSPS	Total (n = 429)	12.5 (7.0)	12.0 (0, 31)
MSNQ	Total (n = 429)	24.1 (13.2)	23.0 (0, 60)
TSQM v1.4	Side effects (n = 353)	78.9 (28.6)	100.0 (0, 100)
	Effectiveness (n = 353)	61.8 (20.0)	67.0 (0, 100)
	Convenience (n = 353)	83.4 (21.6)	94.0 (0, 100)
	Global satisfaction (n = 353)	60.0 (24.5)	57.0 (0, 100)
HADS	Anxiety (n = 429)	8.1 (4.4)	8.0 (0, 19)
	Depression (n = 429)	5.6 (4.0)	5.0 (0, 20)
*Single-item PRO scores*			
PDDS	(n = 429)	2.2 (2.0)	2.0 (0, 7)
HRPQ-MS v2	Lost work absenteeism household (n = 429)	2.3 (3.8)	0.0 (0, 25)
	Lost work absenteeism workplace (n = 218)	1.5 (5.4)	0.0 (0, 40)
	Lost work presenteeism household (n = 429)	2.1 (4.1)	1.0 (0, 60)
	Lost work presenteeism workplace (n = 218)	3.1 (5.0)	0.0 (0, 32)
	Lost work total household (n = 429)	4.4 (6.5)	2.0 (0, 60)
	Lost work total workplace (n = 218)	4.6 (7.9)	0.0 (0, 40)
	Percent lost work absenteeism household (n = 429)	22.0 (29.4)	0.0 (0, 100)
	Percent lost work absenteeism workplace (n = 218)	4.8 (16.5)	0.0 (0, 100)
	Percent lost work presenteeism household (n = 429)	18.9 (22.7)	13.0 (0, 100)
	Percent lost work presenteeism workplace (n = 218)	9.3 (14.7)	0.0 (0, 80)
	Percent lost work total household (n = 429)	41.0 (38.3)	33.0 (0, 100)
	Percent lost work total workplace (n = 218)	14.0 (23.1)	0.0 (0, 100)
	Work presenteeism household (n = 429)	8.6 (8.5)	6.0 (0, 60)
	Work presenteeism workplace (n = 218)	32.8 (13.8)	40.0 (0, 60)

^a^Includes Asian or Pacific Islander, American Indian, and other race. ^b^n = 433 (1 missing patient). ^c^n = 422 (12 missing patients). Any missing data were included in the calculation of percentages

*FSMC* Fatigue Scale for Motor and Cognitive Functions; *HADS* Hospital Anxiety and Depression Scale; *HRPQ-MS v2* Health-Related Productivity Questionnaire in Multiple Sclerosis version 2; *MS* Multiple sclerosis; *MSIS-29 v2* Multiple Sclerosis Impact Scale 29 items version 2; *MSNQ* Multiple Sclerosis Neuropsychological Screening Questionnaire; *MSPS* Multiple Sclerosis Performance Scale; *PDDS* Patient-Determined Disease Steps; *PRO* Patient-reported outcome; *SD* Standard deviation; *TSQM v1.4* Treatment Satisfaction Questionnaire with Medication version 1.4

Baseline PRO scores suggested that participants typically had moderate physical and psychological impact and symptoms, low anxiety and depression, and low satisfaction with prestudy treatment (Table 2). Participants reported moderate impact on their ability to perform daily activities and to remain in the workplace (Table 2).

### Evaluation of the association of the PRO scores with the possible anchor variables

Correlations between each anchor variable and the corresponding PRO score at baseline are shown in Table 3. Overall, high correlations (> 0.7) between the anchor variable and corresponding PRO score at baseline were observed, except for PDDS and MSIS-29 v2 psychological impact score, for which correlation coefficient was moderate (0.38).

**Table 3:** Correlation coefficients between the anchor variables and the corresponding PRO scores at baseline

Score	N	Anchor variables		
		PDDS^a^	MSPS Fatigue item^a^	MSPS Cognition item^a^
MSIS-29 v2 physical impact score	429	**0.81**	–	–
MSIS-29 v2 psychological impact score	429	0.38	–	–
MSPS total score	429	**0.76**	–	–
FSMC physical fatigue score	429	–	**0.80**	–
FSMC cognitive fatigue score	429	–	**0.72**	–
FSMC total score	429	–	**0.78**	–
MSNQ total score	429	–	–	**0.75**

*FSMC* Fatigue Scale for Motor and Cognitive Functions;* MSIS-29 v2* Multiple Sclerosis Impact Scale 29 items version 2;* MSNQ* Multiple Sclerosis Neuropsychological Screening Questionnaire;* MSPS* Multiple Sclerosis Performance Scale;* PDDS* Patient-Determined Disease Steps;* PRO* Patient-reported outcome

^a^Spearman’s rank-order correlation coefficient. Values in bold represent correlation coefficients of > 0.7

Lower correlations were observed between the change in anchors and change in corresponding PRO scores from baseline to Month 12. Overall, correlations were below 0.3 (Additional file 1: Table S1), indicating low correlations between change captured by anchors and change in PRO scores. Only change in MSPS total score and change in PDDS were moderately correlated (0.31).

### Meaningful within-individual improvements for PRO scores

Results for the instruments assessed by anchor-based and distribution-based with triangulation of results for meaningful improvement in PRO scores are summarized in Table 4. The supportive data on meaningful within-individual change for those PRO instruments where only distribution-based methods were possible, since no anchor variable was available (multi-item and single-item scores), are shown in Table 5; the recommended value was defined by 0.5SD at baseline, and the conservative value (multi-item scores only) was based on minimum detectable change. The responder analysis, based on the number of participants with an improvement in score from baseline greater than the clinically meaningful within-individual improvement, was calculated for all PRO scores and is summarized in Additional file 1: Fig. S2. The proportion of responders ranged from 9.1% (HADS Depression) to 34.2% (MSIS-29 v2 psychological impact). The proportion of nonresponders ranged from 52.3% (TSQM effectiveness) to 76.5% (FSMC physical fatigue). Between 5.4% and 23.8% of responses were missing, depending on PRO.

**Table 4:** Within-individual estimates from anchor-based and distribution-based methods for MSIS-29 v2, FSMC, MSPS, and MSNQ scores

Score	Anchor-based methods				Distribution-based methods				Triangulation		
	Anchor	Category	Means^a^	ROC^b^	SD_BL_	0.5 SD_BL_	SEM^c^	Min detectable change	Step^d^	Recommended value^e^	Conservative value^f^
*MSIS-29 v2*											
Physical impact	PDDS	Improvement of 1 category versus No change	− 6.86	− 3.33	25.37	12.69	4.85	13.45	1.67	**− 6.68**	**− 15.03**
		Improvement of 2 categories versus Improvement of 1 category or No change	− 4.09	− 5.00							
		Improvement of 1 or 2 categories versus No change	− 6.57	− 3.33							
Psychological impact	PDDS	Improvement of 1 category versus No change	− 7.30	− 3.70	24.81	12.40	7.20	19.94	3.70	**− 7.40**	**− 22.20**
		Improvement of 2 categories versus Improvement of 1 category or No change	− 5.12	− 7.41							
		Improvement of 1 or 2 categories versus No change	− 7.21	− 3.70							
*FSMC*											
Physical fatigue	MSPS Fatigue item	Improvement of 1 category versus No change	− 1.96	0.00	11.10	5.55	2.37	6.48	1.00	**− 3.00**	**− 7.00**
		Improvement of 2 categories versus Improvement of 1 category or No change	− 2.01	− 3.00							
		Improvement of 1 or 2 categories versus No change	− 2.07	− 1.00							
Cognitive fatigue	MSPS Fatigue item	Improvement of 1 category versus No change	− 2.00	2.00	11.19	5.60	2.37	6.56	1.00	**− 2.00**	**− 7.00**
		Improvement of 2 categories versus Improvement of 1 category or No change	1.24	3.00							
		Improvement of 1 or 2 categories versus No change	− 1.56	2.00							
Total score	MSPS Fatigue item	Improvement of 1 category versus No change	− 3.96	0.00	21.62	10.81	3.47	9.62	1.00	**− 4.00**	**− 10.00**
		Improvement of 2 categories versus Improvement of 1 category or No change	− 0.78	1.00							
		Improvement of 1 or 2 categories versus No change	− 3.63	0.00							
*MSPS*											
Total	PDDS	Improvement of 1 category versus No change	− 1.77	− 1.00	7.03	3.51	2.78	7.71	1.00	**− 2.00**	**− 8.00**
		Improvement of 2 categories versus Improvement of 1 category or No change	− 1.64	− 1.00							
		Improvement of 1 or 2 categories versus No change	− 1.86	− 1.00							
*MSNQ*											
Total	MSPS Cognition item	Improvement of 1 category versus No change	− 2.28	− 1.00	13.24	6.62	2.75	7.62	1.00	**− 3.00**	**− 8.00**
		Improvement of 2 categories versus Improvement of 1 category or No change	− 3.26	− 11.00							
		Improvement of 1 or 2 categories versus No change	− 2.47	− 1.00							

^a^Means refer to the difference in mean change in PRO score between the two categories of the dichotomized anchor variable. ^b^ROC refers to the cut-off in the ROC curve, being the change in PRO score that best discriminates the two categories of the anchor (smallest sum of squares of 1-sensitivity and 1-specificity). ^c^SEM based on Cronbach's alpha calculated at baseline. ^d^Step refers to the distance between two consecutive observable values of the PRO score. ^e^The recommended value for meaningful within-individual improvement was defined from the comparison of mean change in PRO scores between participants showing improvement of one or two categories in the anchor variable and those who did not show any change. ^f^ “Conservative” value was defined based on distribution-based method results

*FSMC* Fatigue Scale for Motor and Cognitive Functions; *MSIS-29 v2* Multiple Sclerosis Impact Scale 29 items version 2; *MSNQ* Multiple Sclerosis Neuropsychological Screening Questionnaire; *MSPS* Multiple Sclerosis Performance Scale; *PDDS* Patient-Determined Disease Steps; *ROC* Receiver-operating characteristic; *SD*_*BL*_ Standard deviation at baseline; *SEM* Standard error of measurement

**Table 5:** Within-individual change estimates from distribution-based methods for TSQM v1.4, HADS, PDDS and HRPQ-MS v2 scores

Score	SD_BL_	0.5 SD_BL_	SEM^a^	Min detectable change	Recommended value^b^
*Multi-item scores*					
TSQM v1.4					
Side effects	28.64	14.32	6.83	18.94	14.32
Effectiveness	19.98	9.99	5.73	15.89	9.99
Convenience	21.62	10.81	5.59	15.48	10.81
Global satisfaction	24.49	12.24	7.72	21.41	12.24
HADS					
Anxiety	4.35	2.18	1.59	4.39	2.18
Depression	4.02	2.01	1.58	4.39	2.01
*Single-item scores*					
PDDS					
﻿–	1.98	0.99	–	–	0.99
HRPQ-MS v2					
Lost work absenteeism household	3.77	1.88	–	–	1.88
Lost work absenteeism workplace	5.35	2.67	–	–	2.67
Lost work presenteeism household	4.07	2.03	–	–	2.03
Lost work presenteeism workplace	5.03	2.51	–	–	2.51
Lost work total household	6.45	3.22	–	–	3.22
Lost work total workplace	7.90	3.95	–	–	3.95
Percent lost work absenteeism household	29.40	14.70	–	–	14.70
Percent lost work absenteeism workplace	16.54	8.27	–	–	8.27
Percent lost work presenteeism household	22.73	11.36	–	–	11.36
Percent lost work presenteeism workplace	14.68	7.34	–	–	7.34
Percent lost work total household	38.33	19.17	–	–	19.17
Percent lost work total workplace	23.13	11.57	–	–	11.57
Work presenteeism household	8.51	4.26	–	–	4.26
Work presenteeism workplace	13.83	6.92	–	–	6.92

^a^SEM based on Cronbach’s alpha calculated at baseline. ^b^The recommended value for meaningful within-individual change was defined by 0.5SD at baseline

*HADS* Hospital Anxiety and Depression Scale; *HRPQ-MS v2* Health-Related Productivity Questionnaire in Multiple Sclerosis version 2; *PDDS* Patient-Determined Disease Steps; *SD*_*BL*_ Standard deviation at baseline; *SEM* Standard error of measurement; *TSQM v1.4* Treatment Satisfaction Questionnaire with Medication version 1.4

## Discussion

This analysis of PRO data from the PROMiS AUBAGIO study has provided candidate values for meaningful within-individual improvement in scores for eight PRO instruments commonly used in people with MS, namely the MSIS-29 v2, FSMC, MSPS, MSNQ, TSQM v1.4, PDDS, HRPQ-MS v2, and HADS (anxiety and depression) instruments. Although not all of these instruments are specific to MS, determination of clinically meaningful within-individual improvement thresholds followed the principles currently recommended by the FDA [15] and resulted from triangulation of results from anchor-based methods, when available, supported by graphical representations of eCDFs of the change in PRO scores groups defined by the change in anchor variables, and distribution-based methods. A systematic approach was applied in this triangulation, with the results from anchor-based methods considered primary, and the full set of results providing a sense of robustness of these results. It is our hope that these values, particularly for the MSIS-29 v2, FSMC, MSPS, and MSNQ scores applying anchor-based methods to provide recommended values, will be helpful in terms of interpreting responses to interventions in the future but also to give better context for previously reported MS studies where PRO data are currently presented.

PRO data are considered of central importance in the assessment of people with MS, and the use of PRO data is likely to become more widespread and clinically important with the development of on-line assessments and smartphone-based technologies that facilitate the exchange of such data between MS patients and their care providers [41]. Furthermore, PROs are often used as secondary or exploratory endpoints in clinical trials, but without a standardized approach, it is difficult to provide a comparative assessment of effectiveness or to enable PRO-based decision-making by regulators or other decision makers. Indeed, a recent literature review has identified inappropriate reporting of PRO data to be a common weakness in MS trial publications and, as a result, an area in need of improvement [42]. To our knowledge, such information was previously only available for certain of the PROs evaluated. For example, the recommended meaningful within-individual improvement for the MSIS-29 v2 physical impact score estimated in our study (− 6.68) is within the range of published values from other studies for worsening and improvement (range of − 4.84 to − 8 based on anchor-based methods, and range of − 2.22 to − 10.4 based on distribution-based methods) [43–45]. We used the PDDS in our anchor-based methods, and previous work using the EDSS as the anchor has reported a meaningful change in MSIS-29 v2 physical impact score of approximately 7.5 for worsening [44, 45] and 5 for improvement [43]. In addition to the MSIS-29 v2 physical impact score, meaningful within-individual improvement value on the MSIS-29 v2 psychological impact score (recommended value of − 7.40) was also estimated, which has not been as widely documented. However, given the wide-ranging impact that MS has on patients' well-being [46], providing meaningful change thresholds on the different subscales broadens the utility of the PRO instruments and allows meaningful improvements in these symptoms to be captured from the patient’s perspective.

Application of minimally important change represents an important tool for enhancing the interpretability of PROs; however to realize the full benefit of the value, an improved understanding is needed particularly around reporting the fundamental properties of the change [47]. To address some of the shortcomings, the FDA are developing a series of methodological patient-focused drug development documents to address how stakeholders can collect and submit patient and caregiver experience data in a stepwise manner for medical product development and regulatory decision-making [16]. Currently available in draft form, the documents aim to provide industry with information to support integration of the patient experience into drug development programs and to guide next steps, such as with external stakeholders who may want to undertake the development of tools within a given disease area [16]. With a more standardized and comparable approach, including providing updated guidance on reliability, validity, and ability to detect minimally important change [48], we hope PROs will contribute more reliably to regulatory decision-making in the future. Providing clinically meaningful thresholds for eight commonly used PRO instruments for MS, albeit requiring some further validation, can only assist with these endeavors in this healthcare space.

Of the eight commonly used PRO instruments selected, some are validated in MS patients [19, 20, 26, 32, 35] and others are studied to various degrees in MS [18, 25, 49–52]. These scales included measures of HRQOL, fatigue, functional performance, work capacity/daily activities, cognitive impairment, anxiety/depression, and satisfaction with treatment, all pertinent to MS symptoms. Performance scales selected had good internal consistency, and most had published data on test–retest reliability. However, PROs used in the PROMiS AUBAGIO study were not exhaustive, and other instruments have been developed, validated, and/or are commonly used in MS, eg, the FSIQ-RMS [53], Neuro-QoL™ [54], SymptoMScreen [55], and FACIT-TS-G [56], among others. Hence, there remains the need for establishing meaningful within-individual change thresholds in a similar manner for these measures, to further support our work. The current study focused on traditionally used instruments that are often applied in the clinical trial or real-world setting, where documenting a meaningful within-individual change in symptoms would be a benefit for people with MS, healthcare providers, and decision makers. The PROMiS AUBAGIO study required enrollees to have fluent English language skills, so we do not know if our observations would be similar for patients completing translated PROs in non-English languages, for which many have been validated. The methods and assumptions used in the current study to determine our observations were based on recommendations from the FDA [15], using triangulation of both anchor-based (primary) and distribution-based (supportive) methods, alongside other considerations highlighted in the literature when calculating clinically meaningful within-individual changes [38, 40, 57]. Although we followed FDA-recommended anchor-based methods, there are no standardized triangulation procedures, to our knowledge. Our triangulation approach involved critically reviewing all estimates from the various methods used, identifying a reference value, then defining a range of values that could be considered “clinically meaningful”. We took this approach to allow estimates for meaningful change for each PRO from the various methods to be considered qualitatively and allow reflection on the variability of the estimates. We did not address questions around different meaningful change values for different subpopulations, as this is an area of debate beyond the scope of this analysis; for example, is meaningful change linked to score only or to context of use? Instead, we took a considered approach, and it is our hope these observations will ultimately facilitate decision-making by regulatory and healthcare authorities moving forwards.

Our study should be considered in light of certain limitations, as the determination of clinically meaningful within-individual change in PRO scores was not a prespecified objective of the PROMiS AUBAGIO study. There is, therefore, some uncertainty associated with the estimated values for meaningful within-individual improvement in all PRO scores based on both anchor- and distribution-based methods. For several PRO scores, no anchor variable assessing the same concept was available; therefore, only distribution-based methods could be applied. For example, values for meaningful change in TSQM v1.4 scores were estimated using distribution-based methods, with estimates of − 10.81 and − 9.99 for the convenience and effectiveness scales, respectively. However, given the high variability in the TSQM these values should be used with caution. The TSQM is commonly used cross-sectionally not longitudinally, although this is not unique to the current study. Although not a valid measure for assessing treatment satisfaction in people with RRMS, scale-to-sample targeting implied that treatment satisfaction may be underestimated by the TSQM; hence, further research is required to overcome this limitation [25]. We therefore recommend the estimated clinically meaningful within-individual improvement values from scales where an acceptable anchor was available (ie, MSIS-29 v2 physical and psychological impact scores; FSMC physical and cognitive fatigue scores, and total score; MSPS total score; MSNQ total score), where both a recommended value and conservative value are provided, and may be useful for sensitivity analyses to allow better interpretation in future studies.

Also of note, the anchor-based estimates were obtained using anchor variables that were not optimal, as indicated by the low correlations of changes in PRO score and anchor variables (Additional file 1: Table S1) and by the overlap observed in eCDFs of change in PRO scores in the categories defined by the anchor variable. This limited discrimination probably led to underestimation of meaningful improvement values. These analyses, especially the anchor-based methods, focused on determination of meaningful within-individual *improvement*; as such there is no certainty that these values could also be used to characterize clinically meaningful within-individual *worsening*. Given MS is generally a disease of declining function, particularly over the long-term, further study is needed to clarify conclusions of clinically meaningful within-individual worsening. We also did not look at how values correlate with physician’s perspectives of clinical importance.

Despite these limitations, our study also had notable strengths. Our study took a broad approach to examine eight commonly used PRO instruments. The size of the study population meant that a reasonably large PRO database was generated, and, given the methods of analysis used, as discussed above, we feel our approach can be considered robust. The study population of people with RRMS was also heterogenous with regard to baseline characteristics, demographics, and baseline PRO scores. Although this introduces inherent variability into our analyses and probably led to higher SDs around the PRO scores (and, thus, overestimated values from distribution-based methods), the heterogenicity of the study population does help to ensure the calculations may be applicable to a broader range of people with RRMS. Our estimates should, therefore, be relevant to a range of future clinical studies in RRMS populations.

## Conclusion

This study has produced recommended estimates for assessing meaningful within-individual improvements based on results from PRO instruments used in MS clinical studies. These estimates will be useful for interpreting improvement scores and communicating the results of future studies evaluating the impact of RRMS on patients and will facilitate decision-making by regulatory and healthcare authorities. Additional work to estimate and confirm meaningful within-individual improvement thresholds in MS target populations, eg, primary-progressive MS (PPMS) and/or nonrelapsing secondary progressive MS (nrSPMS) patients is needed. In addition, work to estimate and confirm meaningful within-individual thresholds for *worsening* scores for all MS patient populations is an area for future research.

## Supplementary Information

**Additional file 1: Fig. S1.** Empirical cumulative distribution functions (eCDFs) for change in PRO scores where anchor variables were available. **A** MSIS-29 v2 physical impact; **B** MSIS-29 v2 psychological impact **C** FSMC Cognitive Score; **D** FSMC Motor Score **E** FSMC Total Score; **F** MSPS Total Score **G** MSNQ. **Fig. S2.** Responders for **A** multi-item and **B** single-item PROs using recommended meaningful within-individual improvement thresholds. **Table S1.** Correlation coefficients between change in anchor variables and the change in the corresponding PRO scores.

## Data Availability

Anonymized data will be shared by reasonable request from any qualified investigator.
