# Supplementary material for: Establishing clinically meaningful within-individual improvement thresholds for eight patient-reported outcome measures in people with relapsing-remitting multiple sclerosis
Source: J Patient Rep Outcomes. 2023 Jul 4;7:61. doi: 10.1186/s41687-023-00594-8 (PMC10319693; doi:10.1186/s41687-023-00594-8)
Supplement: Supplementary file 1 — Additional file 1: Fig. S1. Empirical cumulative distribution functions (eCDFs) for change in PRO scores where anchor variables were available. A MSIS-29 v2 physical impact; B MSIS-29 v2 psychological impact C FSMC Cognitive Score; D FSMC Motor Score E FSMC Total Score; F MSPS Total Score G MSNQ. Fig. S2. Responders for A multi-item and B single-item PROs using recommended meaningful within-individual improvement thresholds. Table S1. Correlation coefficients between change in anchor variables and the change in the corresponding PRO scores. [file 41687_2023_594_MOESM1_ESM.docx]

# Supplementary material

**Establishing Clinically Meaningful Within-Individual Improvement Thresholds for Eight Patient-Reported Outcome Measures in People with Relapsing-Remitting Multiple Sclerosis**

### Figure S1: Empirical cumulative distribution functions (eCDFs) for change in PRO scores where anchor variables were available. (A) MSIS-29 v2 physical impact; (B) MSIS-29 v2 psychological impact (C) FSMC Cognitive Score; (D) FSMC Motor Score (E) FSMC Total Score; (F) MSPS Total Score (G) MSNQ


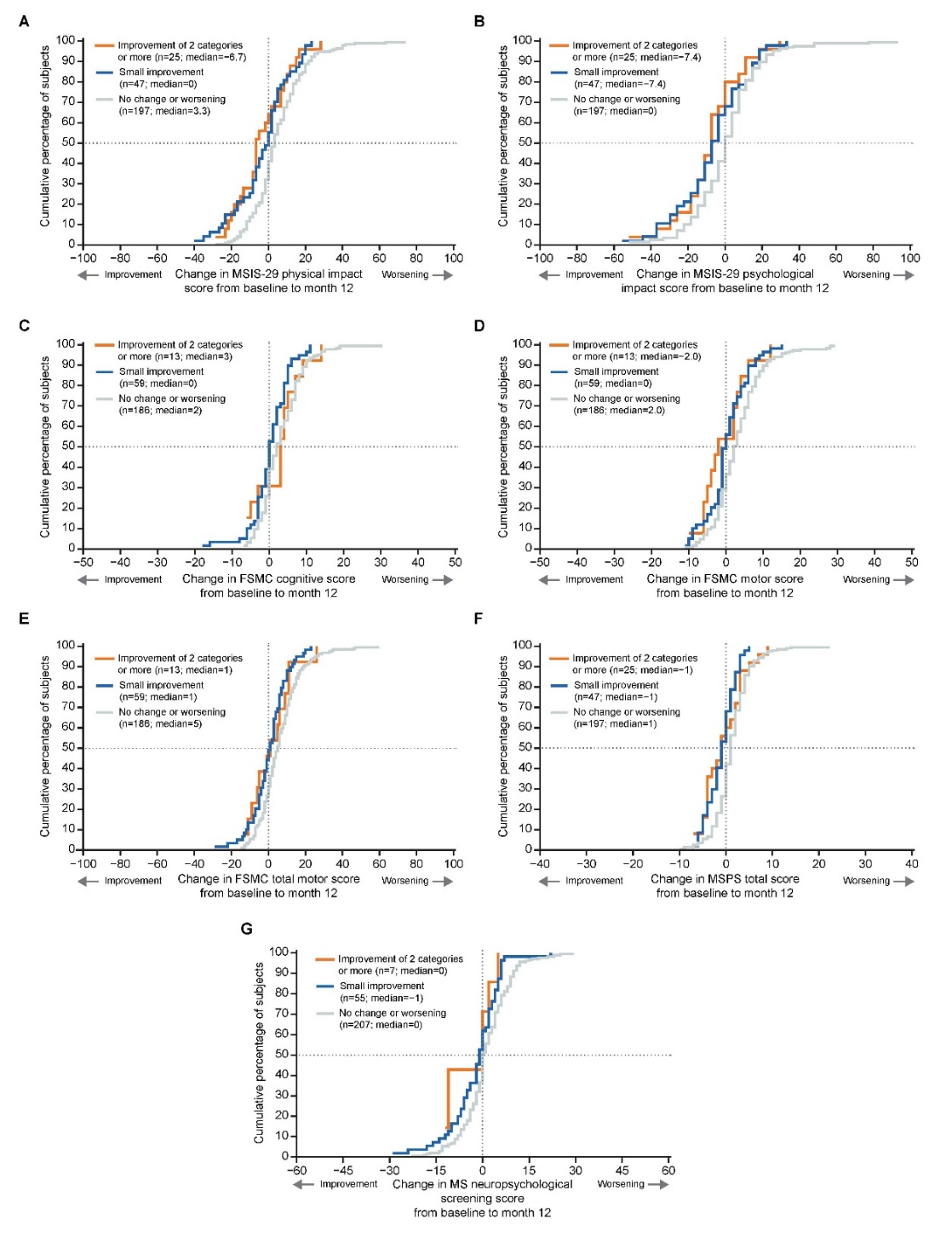


FSMC, Fatigue Scale for Motor and Cognitive Functions; MSIS-29 v2, Multiple Sclerosis Impact Scale 29 items version 2; MSNQ, Multiple Sclerosis Neuropsychological Screening Questionnaire; MSPS, Multiple Sclerosis Performance Scale; PDDS, Patient-Determined Disease Steps; PRO, patient-reported outcome

### Figure S2. Responders for (A) multi-item and (B) single-item PROs using recommended meaningful within-individual improvement thresholds


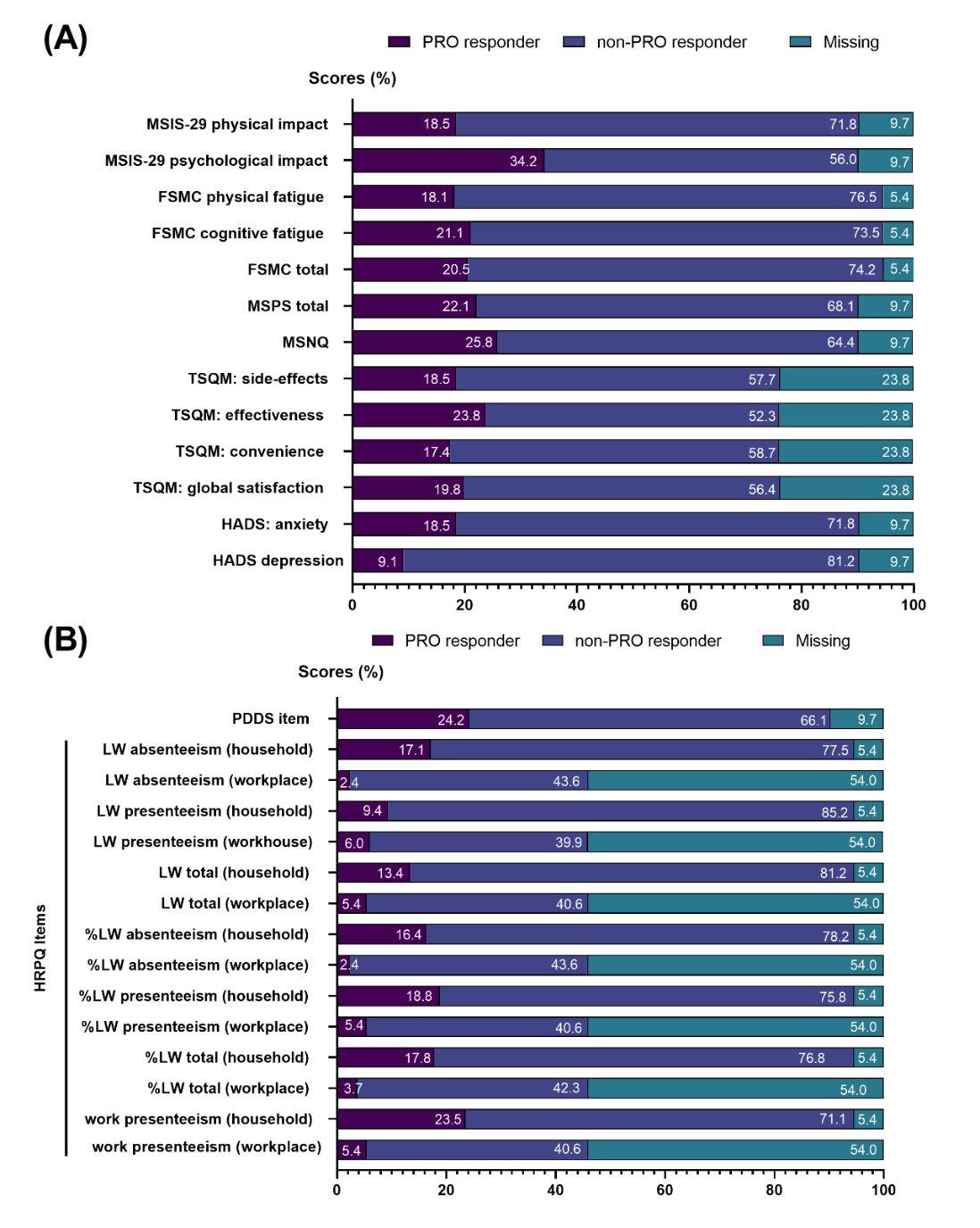


The percentage of responders was calculated at Month 10 for TSQM v1.4 scores, at Month 11 for FSMC and HRPQ-MS v2 scores, and at Month 12 for all other PRO scores, for the recommended PRO responder definition. FSMC, Fatigue Scale for Motor and Cognitive functions; HADS, Hospital Anxiety and Depression Scale; HRPQ, Health-Related Productivity Questionnaire in Multiple Sclerosis version 2; LW, lost work; MSIS-29 v2, Multiple Sclerosis Impact Scale 29 items version 2; MSNQ, Multiple Sclerosis Neuropsychological Screening Questionnaire; MSPS, Multiple Sclerosis Performance Scale; PDDS, Patient-Determined Disease Steps; PRO, patient-reported outcome; TSQM v1.4, Treatment Satisfaction Questionnaire with Medication version 1.4.

### Table S1. Correlation coefficients between change in anchor variables and the change in the corresponding PRO scores^a^

| **Score** | **N** | **Anchor variables** | | |
| --- | --- | --- | --- | --- |
|  |  | **Change in PDDS^b^** | **Change in MSPS Fatigue item^b^** | **Change in MSPS Cognition item^b^** |
| Change in MSIS-29 v2 physical impact score | 269 | 0.27 | - | - |
| Change in MSIS-29 v2 psychological impact score | 269 | 0.18 | - | - |
| Change in MSPS Total score | 269 | 0.31 | - | - |
| Change in FSMC Cognitive score | 282 | - | 0.20 | - |
| Change in FSMC Physical score | 282 | - | 0.26 | - |
| Change in FSMC Total score | 282 | - | 0.25 | - |
| Change in MSNQ Total score | 269 | - | - | 0.25 |

^a^From baseline to Month 11 for FSMC scores, to Month 12 for all other scores; ^b^Spearman rank-order correlation coefficient. FSMC, Fatigue Scale for Motor and Cognitive functions; MSIS-29 v2, Multiple Sclerosis Impact Scale 29 items version 2; MSNQ, Multiple Sclerosis Neuropsychological Screening Questionnaire; MSPS, Multiple Sclerosis Performance Scale; PDDS, Patient-Determined Disease Steps; PRO, patient-reported outcome.
